# The multidimensionality of masculine norms in east Zimbabwe: implications for HIV prevention, testing and treatment

**DOI:** 10.1097/QAD.0000000000002041

**Published:** 2018-12-10

**Authors:** Rebecca Rhead, Morten Skovdal, Albert Takaruza, Rufurwokuda Maswera, Constance Nyamukapa, Simon Gregson

**Affiliations:** a Department of Infectious Disease Epidemiology, Imperial College London School of Public Health London; b Institute of Psychiatry, Psychology and Neuroscience, King's College London, London, UK; c Department of Public Health, University of Copenhagen, Copenhagen, Denmark; d Biomedical Research and Training Institute, Harare, Zimbabwe.

**Keywords:** HIV treatment, masculine social norms, masculinity, Zimbabwe

## Abstract

**Background:**

Research and intervention studies suggest that men face challenges in using HIV services in sub-Saharan Africa. To address these challenges, quantitative measurements are needed to establish the individual-level determinants of masculine norms and their implications for HIV prevention and treatment programmes.

**Methods:**

Survey questions for four masculine norms identified in qualitative research were included in a general-population survey of 3116 men in east Zimbabwe, 2012–2013. Two sets of regression analyses were conducted in an structural equation modelling framework to examine: which sociodemographic characteristics were associated with high scores on each masculinity factor; and how high scores on these masculinity factors differed in their associations with sexual risk behaviour and use of HIV services.

**Findings:**

Sociodemographic characteristics associated with high factor scores differed between masculine norms. In HIV-negative men, more men with scores exceeding one standard deviation above the mean (high scorers) for antifemininity than men with scores under one standard deviation below the mean (low scorers) took steps to avoid infection (61 versus 54%, *P* < 0.01). Fewer high than low scorers on social status reported a recent HIV test (69 versus 74%, *P* = 0.04). In HIV-positive men, more high scorers on sex drive had been diagnosed (85 versus 61%, *P* = 0.02), were on antiretroviral treatment (91 versus 62%, *P* = 0.04), and were in AIDS groups (77 versus 46% *P* = 0.03).

**Conclusion:**

HIV treatment, prevention programmes looking to engage men must consider the multidimensionality of masculine norms. The scale developed in this study is robust and can be used by other large multipurpose surveys to examine masculine social norms.

## Introduction

Masculine social norms have long been recognized as one of the primary factors shaping the patterns of sexual risk behaviour that drive generalized HIV epidemics in sub-Saharan Africa [1,2]. Masculinity is also considered to be a major influence on use of HIV prevention, testing and treatment services. Men, compared with women, have lower levels of HIV testing and receipt of results [3–6], are more likely to delay treatment initiation [7–9], and less likely to be retained in care [10,11], resulting in greater AIDS-related mortality amongst men [12–14].

Masculinity is known to be a multidimensional construct [15–17]. Furthermore, different dimensions of masculinity may differ in the direction and strength of their effects on sexual risk behaviour and use of HIV control services. A clear understanding of these different dimensions, of their determinants, and of their effects in different social contexts could lead to major advances in HIV control by informing and strengthening efforts to address unhelpful norms whilst encouraging and harnessing those that support HIV control [18,19]. Qualitative studies have provided valuable in-depth descriptions of masculine social norms and their influence in selected high HIV prevalence settings [20–26]. These studies suggest that masculine norms may vary in their nature, prevalence, strength and effects between geographical, social and temporal contexts. Repeated measurements in large-scale standardized general-population surveys would permit comparisons across and within countries over time, confirming earlier qualitative findings, and informing efforts to tailor interventions to local contexts and target appropriate population sub-groups. This would also provide quantitative data to aid evaluations of the impact of such interventions.

However, to make this possible, a simplified model describing the main dimensions of masculinity is needed, together with short sets of questions that can be used to measure these dimensions in population surveys. Though there have been earlier attempts to quantitatively measure different dimensions of masculinity, these have predominantly been small-scale studies, which have included a large number of items in their questionnaire [1]. Using a scale with many items is not always feasible for large multipurpose surveys, which require short concise scales to gather data from a wide range of topics while achieving adequate response rates.

We draw on earlier qualitative research and existing measurements of masculine norms to identify and develop basic dimensions and survey questions suitable for our context. We pilot these questions in a general-population survey in the study areas where the qualitative research was done. We analyse the survey data: to identify the sociodemographic determinants of masculine social norms in the study population; and to measure and compare the directions of their associations with sexual risk behaviour and use of HIV prevention, testing and treatment services. Finally, we discuss the plausibility of the results and the possible ways in which findings of the kind obtained in this study could be used to strengthen local HIV control programmes.

## Methods

Data for this study were taken from the Manicaland HIV/STD Prevention Project (Manicaland study) [27]. The Manicaland study is an open-cohort general-population survey, which examines the dynamics of HIV transmission and its impact in eight sites in Manicaland province in eastern Zimbabwe (http://www.manicalandhivproject.org/). These sites represent four of the main socioeconomic strata in Manicaland: small towns, agricultural estates, roadside settlements and subsistence farming areas. Topics covered in individual interviews included socioeconomic characteristics, sexual behaviour, psychosocial characteristics and use of HIV testing and treatment services. Participants were also requested to provide a dried blood sample (DBS) for HIV sero-testing. All participants gave informed consent to participate and are free to withdraw from the study at any time. Data gathered from participants has been anonymized to ensure that they cannot be identified. Respondents’ names are not recorded but to permit subsequent linking of the data in the study database with laboratory results, each participant is assigned a study site number, a household number and a unique reference number. In this article, we analysed data from 3116 men who participated in round six (2012–2013) of the Manicaland study.

### Measuring masculine social norms

The measures used to capture masculine norms were developed in an iterative process of consulting qualitative research conducted in the same study areas in 2010 [25,28], the existing literature on measures of masculine norms and local stakeholders. After a review of key literature on the measurement of masculine norms [29–32] we revisited the qualitative data to explore the relevance of the various dimensions of masculine norms framing existing measurements. This process led us to focus on four prominent dimensions of masculine norms, which seemed particularly pertinent to our qualitative material. Specifically, we adopted ‘toughness,’ ‘antifemininity,’ and ‘social status’ from Thomson and Pleck [31] and ‘sex drive’ from Luyt [29]. In our context, and in the study of masculine norms in high HIV prevalence communities, we define the four dimensions of masculine norms as follows:

(1) Toughness – How a man perceives his physical resilience and strength.
(2) Antifemininity – How a man distances himself from feminine roles and spaces.
(3) Sex drive – How a man expresses his sexuality in the context of HIV.
(4) Social status – How a man deals with his associations with HIV within his community.

This latter masculine norm – social status – relates to the ways in which men believe they should perceive and be perceived by other men in relation to HIV and illness, but also how they should deal with HIV more generally. Dimensions of social norms that were not included because of little resonance with our qualitative data included amongst others homophobic attitudes, self-reliance or independence and aggression.

To develop measures of each of these dimensions, statements in the qualitative data, expressing a masculine social norm, were extracted. The process of picking, rephrasing and clustering the statements into the four dimensions of masculine social norms was iterative and was done with reference to lessons learned from the Male Attitude Norms Inventory-II, which has been tested in neighbouring South Africa [29] and guidance from field staff in Manicaland. This resulted in 16 newly designed masculinity measures that were included in round six of the Manicaland study (each representative of one of the four masculine social norms). The final list of questionnaires is shown in Table 1.

### Data analysis

**Fig. 1:**
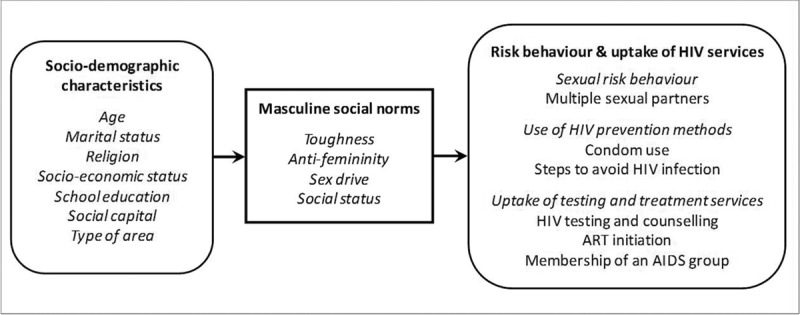
Theoretical framework for the individual-level socio-demographic determinants of masculine social norms, and the influence of masculine social norms on (i) sexual risk behaviour, (ii) use of HIV prevention, and (iii) uptake of testing and treatment services amongst men in east Zimbabwe.

We propose that the adoption of masculine social norms is influenced by sociodemographic characteristics and that these norms, in turn, have different effects on sexual risk behaviour for HIV acquisition, use of HIV prevention methods and use of HIV testing and treatment services (Fig. 1). However, we have no a priori assumptions regarding the strength or direction of these associations. In that sense, this is predominantly an exploratory assessment of how each masculine norm is associated with sociodemographic characteristics and HIV-related behaviours. Indeed, the purpose of this article is to establish what the dimensions are and demonstrate that they have different determinants and effects.

Data analysis was done using confirmatory factor analysis (CFA) and regression in a structural equation modelling (SEM) framework. CFA is a type of SEM that deals specifically with measurement models – the relationships between observed indicators and latent variables [33]. In our study, the items listed in Table 1 are used to produce four factors representing each masculine social norm (‘toughness,’ ‘antifemininity,’ ‘sex drive’ and ‘social status’). Each of these factors exists as a continuous variable, which reflects the extent to which our survey participants endorse that particular masculine norm (i.e. participants who strongly endorse a particular norm will have a high score for the relevant factor).

Once a well fitting factor model is specified (for more details on this, see Supplementary Material), two sets of regression analysis are conducted in an SEM framework where our factors are generated and simultaneously either treated as an independent variable (IV) or a dependent variable, respectively. This allows us to (a) examine, which sociodemographic measures (independent variable) are associated with high scores on each masculinity factor (dependent variable), and (b) how scoring highly on masculinity factors (independent variable) is associated with risky behaviours and use of HIV services (dependent variable). Our examination of the latter was stratified by HIV status as some HIV services are only applicable to HIV-positive participants. HIV status of all participants is determined through DBS testing.

All analyses were performed in R using Lavaan – an R Package for structural equation modelling [34].

### Sociodemographic determinants of masculinity

Age, marital status, religion, socioeconomic status, school education, social capital and residential were considered as potential individual-level determinants of adoption of masculine social norms. Participants’ ages were divided into four sub-groups (15–24; 25–34; 35–44; 45–54). Participants’ marital status was categorized as married (formally married or co-habiting), widowed, divorced/separated or never married. For religious denomination, we used Manzou's four category grouping of Manicaland churches [13]. For socioeconomic status (SES), we used a continuous combined measure of sellable and nonsellable assets [35], divided into terciles (1 = poorest → 3 = richest). A binary measure of education was used to distinguish between participants educated up to primary level, and those educated to a secondary or higher level of education. Social capital was defined as the number of well rated community groups (excluding church groups and AIDS groups) that a participant belongs to [36,37]. Finally, participants were categorized by whether they resided in towns, agricultural estates, roadside settlements or subsistence farming areas.

### Sexual risk behaviour and uptake of HIV prevention, testing and treatment services

#### Sexual risk behaviour

Sexual risk behavior was assessed using self-reports of numbers of sexual partners in the 3 years prior to interview. Having more than one concurrent partner at the time of interview and having had any nonregular partners in the 3 years prior to interview were included in the analysis as binary measures.

#### Use of HIV prevention methods

Recent condom use and the adoption of strategies to avoid HIV infection were taken to represent HIV prevention. A binary measure of recent condom use was used, which captured whether participants used a condom throughout their most recent sex act prior to interview. Whether participants (or their spouse/partner) were taking steps to ‘avoid HIV and AIDS?’ was included as a binary variable.

#### Uptake of HIV testing and treatment services

Testing behaviour was measured using two binary variables: whether participants had been tested for HIV in the past 3 years; and whether they had been tested in their lifetime.

HIV diagnosis, treatment initiation and AIDS group membership were assessed as outcomes only in HIV-positive men. Participants were considered as having been diagnosed if they were HIV-positive in independent serotesting conducted for research purposes only (a free parallel voluntary counselling and testing service was provided for those wishing to know their infection status) using the DBS specimens collected in the survey, and in addition, reported that the result of their recent HIV test was positive. Participants were considered to have been initiated onto antiretroviral treatment for HIV if they reported taking drugs ‘that stop HIV from causing AIDS.’ AIDS group membership was used a binary measure of whether HIV-positive men were a member of a nearby AIDS support group.

## Results

### Statistical model structure

A four factor CFA model was analysed and produced borderline goodness-of-fit statistics (CFI: 0.816, TLI: 0.775, RMSEA: 0.065, SRMR: 0.111). Small modifications were made to the model based on the modification indices to improve model fit with without severely detract from the hypothesised framework. However, these modifications still failed to produce satisfactory goodness-of-fit. Separate individual CFA models were then produced for each factor, these achieved excellent goodness-of-fit scores and were found to be metric invariant across age-group and residential area type (see Supplementary Material for details).

### Sociodemographic determinants of masculinity

Table 2 presents a series of SEM regression models, where sociodemographic variables are individually regressed onto a single masculine norm factor and each masculinity factor is modelled as a continuous latent variable. Table 2 also displays results of multivariable analysis where covariates significantly associated with the relevant outcome at *P* < 0.1 are included in the model. All models in Table 2 are adjusted for age group.

The different masculine norms were associated with different sociodemographic characteristics and, in some instances, had different directions of association (Table 2). Toughness was more common in men living on agricultural estates and in subsistence farming areas (than in those living in towns), in men who participated in community groups, and in men from the poorest quartile of households; but was less common in married, separated and divorced men (than in never married men), in men with greater school education and in men from Christian churches (than in those with no religion). Antifemininity was less common in towns (than in agricultural estates, roadside settlements and subsistence farming areas) and more common in currently married, divorced and separated men (than in never married men). Like toughness, antifemininity showed a positive association with community participation; but there was no association with education level, a negative association with Spiritual church membership, and a positive association with living in the least poor quartile of households. Sex drive also was more common in currently married, separated and divorced (and also in widowed) men, men living in towns (than in all other types of areas) and in men in community groups; but tended to be less common (*P* = 0.06) in more educated men. Similar to toughness – but unlike antifemininity – sex drive showed a negative association with membership of a Christian church. As with each of the other three masculine social norms, social status was positively associated with community participation. Similar to toughness, social status showed a negative association with greater education; and similar to antifemininity and sex drive, a positive association with residence in a subsistence farming area (versus living in a town). No associations were found with household wealth or church denomination and no results could be obtained for presence or otherwise of an association between social status and marriage. None of the masculine social norms was associated with HIV infection status. These associations remain unchanged in the multivariable models.

Sexual risk behaviours and use of HIV prevention methods, HIV testing and antiretroviral treatment

Table 3 shows the predicted probabilities of experiencing each outcome for high and low (masculine social norm) factor scorers. The difference in predicted probability between low (−1 standard deviation) and high (+1 standard deviation) factor scorers is also shown in the right-hand column for each masculine norm.

Amongst HIV-negative men, high scorers on the Antifemininity factor had a higher probability of taking steps to avoid HIV than low scorers (61 versus 54%, *P* < 0.01). High scorers on the sex drive factor had a higher probability of reporting steps to avoid HIV infection (61 versus 54%, *P* < 0.01), and also of having been tested – both in the last 3 years (75 versus 69%, *P* = 0.02) and in their lifetime (82 versus 74%, *P* < 0.01). Finally, high scorers on the social status factor had a borderline statistically significant increased probability of recent condom use (21 versus 15%, *P* = 0.05), and a lower probability of having been tested for HIV recently (69 versus 74%, *P* = 0.04). No significant (*P* < 0.05) associations were found between the toughness factor and any of the outcomes in the study. Also, no associations were found between masculine social norms and reported sexual risk behaviour.

Amongst HIV-positive men, sex drive was the only masculine norm significantly associated with any of the outcomes. High scorers on this factor were more likely than low scorers to have been diagnosed for HIV infection (85 versus 61%, *P* = 0.02), to be on antiretroviral treatment (91 versus 62%, *P* = 0.04), and to be a member of an AIDS group (77 versus 46% *P* = 0.03).

## Discussion

A better understanding of the multidimensional nature of masculinity, together with methods for measurement of its principal dimensions in general population surveys, is needed urgently to provide a basis for designing more effective interventions to end the major HIV epidemics in sub-Saharan African populations. Here we developed and piloted a new module of 16 survey questions to measure four different dimensions of masculine social norms. The results provide some of the first quantitative measurements of these norms, their individual-level determinants and their associations with key outcomes for HIV control. The findings provided support for our hypotheses that different masculine social norms are shaped by different individual characteristics and, also, that these different norms differ in their associations with HIV-related outcomes. Key findings for individual dimensions of masculinity include contrasting directions of effect of marriage on toughness (a negative association) and antifemininity and sex drive (positive associations); HIV-negative men with low sores for antifemininity being less likely than those with high scores to have taken steps to avoid HIV infection – despite their having similar patterns of sexual risk behaviour; and men with low scores for sex drive being less likely to have taken steps to avoid HIV (if HIV-negative), less likely to have had a recent HIV test, and less likely to have been diagnosed, to be on treatment and to be in an AIDS support group (if HIV-positive).

The small size of this 16-item scale and its ability to capture such concepts is potentially very valuable. Other large multipurpose surveys can implement this scale in different settings, allowing others to further unpick multidimensional masculinity and its associations with HIV-related behaviours, thus opening the door for future studies. Furthermore, the value of this scale may extend beyond the field of HIV allowing masculine norms to be assessed in relation to other health issues, possibly with minor adaptations if the Social Status dimensions needs to be replaced with something less HIV-specific.

Though we made no assumptions regarding the strength or direction of the associations between this study's masculine norms either sociodemographic or HIV-related behaviours, it was surprising to find that there was no association between scoring highly on the Sex drive social norm and engaging in risky sexual behaviour (regardless of HIV status). We can speculate that this may be because those who score highly on this social norm, rather than behave recklessly, are aware of the consequence of endorsing such a norm and act responsibly, though further qualitative research is needed to help us understand these associations.

The plausibility of our findings is supported by the goodness-of-fit score achieved by each of the separate factor models. Though a four-factor model achieved more mediocre scores, it is not uncommon to see a slight decrease in goodness-of-fit when increasing the size of a model. Introducing additional variables and parameters places constraints on the model, increasing its complexity and making it harder to fit the data well [38]. Furthermore, our findings are consistent with those from previous qualitative studies.

Strengths of the study include its large general population sample and use of measures of masculine social norms derived from qualitative research conducted in the same study areas. Limitations include the use of cross-sectional data, which makes it difficult to establish the directions of causality for observed associations. An informal confidential voting interview procedure was used to reduce social desirability bias in self-reported data on sexual risk behaviour and condom use; however, residual bias in these reports may explain the lack of associations found between dimensions of masculinity and these outcomes.

The study was conducted across four different socioeconomic strata in east Zimbabwe and, taken together with findings from studies elsewhere [2,19,21,29], our results suggest that the dimensions of masculinity examined in this article are generalizable to other parts of sub-Saharan Africa. Further qualitative research is needed to refine and to explore the generalizability of the specific questions used in this study.

Our use of cross-sectional data limits the current study's ability to provide a life course perspective. However, it does highlight how specific individual and social determinants, which are temporally specific, shape social norms. The results displayed in Table 2 demonstrate that men from different age groups, marital status and locations (Manicaland residents are highly mobile [28]) are associated with different social norms. These findings hint that what it means to ‘be a man’ is constantly under negotiation, over time and across space. Follow-up and longitudinal analysis of data from participants in this survey may allow us to determine if the strength and direction of the associations found in this article vary over the life course.

The results of this analysis highlight the power of social and structural forces in determining how masculine norms come to shape HIV risk behaviours and engagement with HIV services. This multidimensional view of masculine norms supports the case for a differentiated HIV response [39] and has important implications for HIV control programmes that seek to address adverse effects of masculinity. The contrasting patterns of masculine social norms found between single and married men indicate that different approaches to HIV control may need to be targeted to men at these different life stages in east Zimbabwe. The positive associations we found between participation in community groups and all dimensions of masculinity are consistent with other findings of negative social capital for men in the same study populations [36,37]; but suggest that community groups may provide an important entry point for interventions to address unhelpful aspects of masculinity. We must heed of these findings and develop safe social spaces where men can steer the emergence, persistence or disappearance of masculine norms that we know shape engagement with HIV services.

## Acknowledgements

Authors’ contribution: R.R., M.S. and S.G. were involved in study concept and design, as well as the design of the analysis. C.N., R.M and A.T. acquired and curated the data. R.R. conducted the statistical analysis supervised by S.G. R.R., M.S. and S.G. interpreted the results and drafted the article.

Funding sources: S.G. thanks the Wellcome Trust for funding (grants: 084401/Z/07/B and 090285MA). The views expressed are those of the authors and not necessarily those of the Wellcome Trust.

The funders had no role in study design, data collection and analysis, data interpretation, decision to publish or writing of the report. The corresponding author had full access to all the data in the study and had final responsibility for the decision to submit for publication.

Data access: Data produced by the Manicaland Project can be obtained from the project website: http://www.manicalandhivproject.org/data-access.html. Here we provide a core dataset, which contains a sample of sociodemographic, sexual behaviour and HIV testing variables from all six rounds of the main survey, as well as data used in the production of recent academic publications. If further data is required, a data request form must be completed (available to download from our website) and submitted to s.gregson@imperial.ac.uk. If the proposal is approved, we will send a data sharing agreement, which must be agreed upon before we release the requested data.

Ethical approval: Prior ethical approval for the Manicaland study was obtained from the Medical Research Council of Zimbabwe (MRCZ/A/681) and the Imperial College Research Ethics Committee (ICREC_9_3_13).

### Conflicts of interest

There are no conflicts of interest.

## Supplementary Material

Supplemental Digital Content

## Figures and Tables

**Table 1:** Masculine social norms and questions used for their measurement in the Manicaland study, Zimbabwe, 2012–2013.

Masculine social norm	Survey question
Toughness	‘Men are strong and therefore less likely to need a doctor’ [STRONG]
	‘Minor illnesses can be fought off if you don’t give in to them’ [ILL]
	‘There is no need to go and see a doctor unless you are very ill’ [DOCTOR]
	‘A man who goes to the hospital is considered weak’ [WEAK]
Antifemininity	‘Men who take sick children to the hospital, or cook at home, should be proud of what they do’ [PROUD]
	‘A man should not go with his partner for antenatal check-ups at the local clinic’ [ANC]
	‘It is appropriate for a woman to be the primary breadwinner of a household’ [BREADWINNER]
	‘Men feel comfortable going to the hospital and no problems seeking help’ [HOSPITAL]
Sex drive	‘Men have a sex drive that needs to be satisfied’ [SEXDRIVE]
	‘A real man enjoys a bit of risk-taking now and then’ [RISK]
	‘Men are always ready for sex’ [READY]
	‘A man should make sure that he knows about HIV’ [HIV]
Social status	‘A man will lose respect if he admits to having HIV’ [RESPECT]
	‘If a man is sick, he should not let others see he is in pain’ [PAIN]
	‘Men get embarrassed if a brother is found to be HIV positive’ [BROTHER]

In the survey, men were asked whether they agreed or disagreed with each of these statements.

**Table 2:** Associations between socio-demographic characteristics and masculine social norms, men aged 15–54 years, Manicaland.

		Toughness				Antifemininity				Sex Drive				Social Status			
		Univariate		Multivariable		Univariate		Multivariable		Univariate		Multivariable		Univariate		Multivariable	
	*N*	Coefficient	*P* value	Coefficient	*P* value	Coefficient	*P* value	Coefficient	*P* value	Coefficient	*P* value	Coefficient	*P* value	Coefficient	*P* value	Coefficient	*P* value
Age (*n* = 3116)																	
15-24	1352	1		1		1		1		1		1		1		1	
25-34	788	−0.06 (−0.14 to 0.01)	0.11	(−0.07 to 0.15)	0.46	0.28 (0.17–0.39)	0.00	−0.03 (−0.14 to 0.09)	0.63	0.55 (0.44–0.67)	0.00	0.08 (−0.07 to 0.24)	0.29	−0.05 (−0.12 to0.02)	0.15	−0.11 (−0.2 to −0.03)	0.01
35-44	599	−0.07 (−0.15 to 0.01)	0.09	0.02 (−0.10 to 0.14)	0.73	0.3 (0.17–0.42)	0.00	−0.05 (−0.18 to 0.08)	0.49	0.59 (0.46–0.71)	0.00	0.07 (−0.1 to 0.24)	0.43	0.03 (-0.04-0.11)	0.42	−0.05 (−0.15 to 0.05)	0.32
45-54	377	0.16 (−0.06 to 0.26)	0.00	0.21 (0.07–0.35)	<0.01	0.48 (0.32–0.63)	0.00	0.13 (−0.02 to 0.28)	0.08	0.67 (0.51–0.82)	0.00	0.17 (−0.03 to 0.37)	0.09	0.13 (0.03-0.22)	0.01	0 (−0.12 to 0.12)	0.99
HIV infection status (*n* = 3116)																	
Infected	381	1		1		1		1		1		1		1		1	
Uninfected	2735	0.01 (−0.08 to 0.11)	0.78	0.03 (−0.07 to 0.13)	0.60	−0.03 (−0.15 to 0.09)	0.65	−0.04 (−0.15 to 0.07)	0.46	0.04 (−0.09 to 0.18)	0.51	(−0.1 to 0.18)	0.61	0.04 (-0.05-0.13)	0.39	0.03 (−0.07 to 0.14)	0.53
Marital status (*n* = 3114)																	
Never married	1408	1		1		1		1		1		1		1		–	–
Currently married	1557	(−0.29 to −0.12)	<0.01	−0.23 (−0.34 to −0.12)	<0.01	0.14 (0.05–0.24)	<0.01	0.16 (0.04–0.27)	0.01	0.71 (0.57–0.84)	<0.01	0.49 (0.34–0.64)	0.00	NA	–	–	–
Separated or divorced	118	−0.20 (−0.35 to −0.04)	0.02	−0.15 (−0.33 to 0.02)	0.09	0.23 (0.05–0.41)	0.01	0.23 (0.03–0.43)	0.02	0.55 (0.28–0.82)	<0.01	0.39 (0.13–0.66)	0.00	NA	–	–	–
Widowed	31	−0.03 (−0.29 to 0.24)	0.84	0.03 (−0.24 to 0.30)	0.82	0.19 (−0.10 to 0.48)	0.20	0.28 (0.04 to 0.61)	0.09	0.49 (0.08–0.91)	0.02	0.37 (−0.01 to 0.75)	0.06	NA	–	–	–
Church denomination (*n* = 3116)																	
No religion	559	1		1		1		1		1		1		1		–	–
Christian church	1440	−0.10 (−0.18 to −0.01)	0.02	−0.11 (−0.20 to 0.02)	0.01	−0.01 (−0.12 to 0.10)	0.85	−0.02 (−0.11 to 0.07)	0.69	−0.12 (−0.24 to −0.01)	0.04	−0.15 (−0.28 to −0.02)	0.02	−0.02 (−0.10–0.06)	0.55	–	–
Spiritual church	727	0.03 (−0.06 to 0.13)	0.51	0.01 (−0.09 to 0.11)	0.87	−0.14 (−0.26 to −0.02)	0.02 (−0.22 to −0.01)	−0.12	0.03 (−0.17 to 0.08)	−0.04 (−0.19 to 0.09)	0.50	−0.05 (−0.02 to 0.16)	0.45	0.07	0.15	–	–
Other church	390	−0.03 (−0.14 to 0.08)	0.62	−0.06 (−0.17 to 0.05)	0.30	−0.02 (−0.17 to 0.12)	0.74	−0.04 (−0.17 to 0.08)	0.48	−0.07 (−0.22 to 0.08)	0.38	−0.09 (−0.25 to 0.07)	0.29	0.02 (−0.08 to 0.12)	0.71	–	–
School education (*n* = 3116)																	
Primary or less	525	1		1		1		–	–	1		1		1		1	
Secondary or higher	2581	−0.32 (−0.40 to −0.23)	<0.01	−0.25 (−0.34 to −0.17)	0.00	−0.04 (−0.15 to 0.08)	0.50	– –	– –	0.11 (0.00–0.23)	0.06	0.06 (−0.05 to 0.18)	0.29	−0.24 (−0.33 to −0.15)	<0.01	−0.29 (−0.39 to −0.19)	0.00
Household wealth (*n* = 2859)																	
First (poorest) quartile	317	1		1		1		1		1		–	–	1		–	–
Second quartile	1192	−0.13 (−0.23 to −0.02)	0.02	−0.12 (−0.22 to −0.01)	0.03	0.02 (−0.11 to 0.15)	0.76	0.03 (−0.08 to 0.15)	0.54	−0.07 (−0.22 to 0.08)	0.35	–	–	−0.04 (−0.14 to 0.06)	0.48	–	–
												–	–			–	–
Third quartile	906	−0.11 (−0.22 to 0.00)	0.04	−0.1 (−0.20 to 0.01)	0.08	0.06 (0.07–0.20)	0.35	0.06 (−0.06 to 0.17)	0.34	0.04 (−0.11 to 0.20)	0.58	–	–	0.00 (−0.10 to 0.10)	0.97	–	–
Fourth quartile	444	−0.22 (−0.35 to −0.09)	<0.01	−0.16 (−0.29 to −0.04)	0.01	0.25 (0.10–0.41)	<0.01	0.17 (0.03–0.3)	0.02	0.08 (−0.10 to 0.25)	0.38	–	–	−0.10 (−0.21 to 0.02)	0.11	–	–
Social capital (*n* = 3116)																	
No community group membership	1466	1		1		1		1		1		1		1		1	
One community group	880	0.08 (0.01–0.15)	0.03	0.11 (0.04–0.19)	0.00	0.11 (0.02–0.20)	0.02	0.07 (−0.01 to 0.15)	0.07	0.12 (0.02–0.22)	0.01	0.07 (−0.03 to 0.17)	0.17	0.13 (0.04–0.21)	<0.01	0.13 (0.05–0.21)	0.00
>1 community group	770	0.13 (0.05–0.21)	<0.01	0.2 (0.12–0.28)	0.00	0.24 (0.14–0.34)	<0.01	0.18 (0.09–0.28)	0.00	0.46 (0.35–0.58)	<0.01	0.4 (0.28–0.51)	0.00	0.23 (0.13–0.32)	<0.01	0.24 (0.15–0.33)	0.00
Study site type (*n* = 3,116)																	
Town	744	1		1		1		1		1		1		1		1	
Agricultural estate	659	0.14 (0.05–0.23)	<0.01	0.13 (0.03–0.22)	0.01	−0.30 (−0.43 to −0.18)	<0.01	−0.2 (−0.31 to −0.1)	0.00	−0.34 (−0.47 to −0.21)	<0.01	−0.27 (−0.4 to −0.14)	0.00	−0.04 (−0.13 to 0.04)	0.29	−0.04 (−0.14 to 0.05)	0.38
Roadside settlement	884	0.04 (−0.04 to 0.13)	0.29	−0.01 (−0.10 to 0.08)	0.78	−0.21 (−0.32 to −0.09)	<0.01	−0.16 (−0.26 to −0.06)	0.00	−0.12 (−0.24 to 0.00)	0.05	−0.07 (−0.19 to 0.05)	0.23	−0.01 (−0.09 to 0.07)	0.77	−0.04 (−0.13 to 0.06)	0.46
Subsistence farming area	829	0.11 (0.02–0.19)	0.01	0.04 (−0.04 to 0.13)	0.33	−0.24 (−0.35 to −0.12)	<0.01	−0.15 (−0.25 to −0.05)	0.00	−0.18 (−0.31 to −0.06)	<0.01	−0.11 (−0.23 to 0.01)	0.08	−0.08 (0.17 to 0.00)	0.04	−0.12 (−0.22 to −0.03)	0.01

95% confidence intervals are shown in parentheses; NA, no results because of small sample size or lack of variation in the outcomes.

**Table 3:** Associations between masculine social norms and sexual risk behaviour and use of HIV services, men aged 15–54 years.

		Toughness			Antifemininity			Sex drive			Social status		
Outcomes		Coefficient	*P* value	Difference between high and low factor scorers	Coefficient	*P* value	Difference between high and low factor scorers	Coefficient	*P* value	Difference between high and low factor scorers	Coefficient	*P* value	Difference between high and low factor scorers
HIV-negative men (*n* = 2735)													
Sexual risk behaviour	*N*												
Concurrent partnerships	48	0.02 (− 0.02 to 0.05)	0.35	0% [51–51%]	0.02 (−0.02–0.05)	0.35	0% [51–51%]	0.00 (−0.02 to 0.02)	0.97	0% [51–51%]	0.01 (−0.02 to 0.03)	0.55	0% [51–51%]
Recent nonregular partner(s)	647	0.01 (−0.05 to 0.07)	0.80	NA	0.04 (−0.04–0.12)	0.32	NA	0.03 (−0.04 to 0.09)	0.40	NA	0.00 (−0.06 to 0.06)	0.99	NA
Use of HIV prevention methods													
Recent condom use	415	0.13 (−0.05 to 0.30)	0.16	4% [20–16%]	0.17 (−0.02 to 0.05)	0.14	4% (20–16%)	0.15 (−0.05 to 0.35)	0.15	3% (19–16%)	0.20 (0.00 to 0.40)	0.05	6% [21–15%]
Taken steps to avoid HIV infection	2306	−0.01 (−0.04 to 0.02)	0.59	−1% [57–58%]	0.14 (0.09–0.20)	<0.01	7% [61–54%]	0.13 (0.09v0.17)	<0.01	7% [61–54%]	0.03 (−0.01 to 0.07)	0.16	1% [58–57%]
Uptake of HIV testing													
HIV test - in lifetime	1530	−0.09 (−0.21 to 0.03)	0.15	−3% [77–80%]	0.04 (−0.16–0.24)	0.67	1% [79–78%]	0.24 (0.11–0.36)	<0.01	8% [82–74%]	−0.10 (−0.25 to 0.05)	0.17	−3% [77–80%]
HIV test - in last 3 years	1376	−0.11 (−0.22 to0.01)	0.08	−5% [69–74%]	−0.03 (−0.23 to 0.18)	0.80	−1% [71–72%]	0.16 (0.03–0.28)	0.02	6% [75–69%]	−0.16 (−0.31 to −0.01)	0.04	−5% [69–74%]
HIV-positive men (*n* = 381)													
Sexual risk behaviour	*N*												
Concurrent partnerships	18	0.00 (−0.04 to 0.04)	0.81	1% [48–47%]	−0.05 (−0.15 to 0.05)	0.36	−1% [47–48%]	−0.03 (−0.15 to 0.08)	0.57	−1% [47–48%]	−0.02 (−0.08 to 0.04)	0.54	−1% [47–48%]
Recent nonregular partner(s)	130	−0.03 (−0.19 to 0.12)	0.68	NA	0.08 (−0.19 to 0.35)	0.57	NA	0.08 (−0.18 to 0.34)	0.55	NA	−0.03 (−0.18 to 0.12)	0.68	NA
Use of HIV prevention methods													
Recent condom use	179	−0.19 (−0.52 to 0.15)	0.28	−7% [63–70%]	−0.24 (−0.68 to 0.19)	0.27	−9% [62–71%]	0.06 (−0.36 to 0.47)	0.79	2% [68–66%]	−0.02 NA	0.35	NA
Taken steps to avoid HIV infection	324	−0.03 (−0.16 to 0.01)	0.63	−1% [51–52%]	0.09 (−0.13 to 0.31)	0.43	3% [53–50%]	0.12 (−0.05 to 0.07)	0.41	5% [54–49%]	−0.08 NA	0.80	NA
Uptake of HIV testing and treatment													
HIV infection status diagnosed	179	0.09 (−0.26 to 0.44)	0.61	3% [76–73%]	0.20 (−0.24 to 0.64)	0.37	5% [77–72%]	0.95 (0.13–1.77)	0.02	24% [85–61%]	0.31 (−0.05 to 0.66)	0.10	NA
Antiretroviral treatment	138	0.14 (−0.21 to 0.48)	0.44	4% [81–77%]	0.20 (−0.27 to 0.67)	0.39	4% [81–77%]	1.45 (0.07–2.83)	0.04	29% [91–62%]	NA	NA	NA
AIDS group member	74	−0.17 (−0.57 to 0.24)	0.42	−7% [59–66%]	0.45 (−0.07 to 0.97)	0.09	15% [70–55%]	0.83 (0.09–1.57)	0.03	31% [77–46%]	NA	NA	NA

Coefficients and differences between high (+1 standard deviation) and low (−1 standard deviation) factor scorers from structural. 95% confidence intervals shown in parentheses; range between 1 SD above and below mean shown in square brackets.
