# Supplementary material for: The multidimensionality of masculine norms in east Zimbabwe: implications for HIV prevention, testing and treatment
Source: AIDS. 2018 Dec 10;33(3):537–46. doi: 10.1097/QAD.0000000000002041 (PMC6365253; doi:10.1097/QAD.0000000000002041)
Supplement: Supplemental Digital Content [file aids-33-537-s001.docx]

**Supplementary Material**

**The multidimensionality of masculine norms in east Zimbabwe: implications for HIV prevention, testing and treatment**

1. **Structural equation modelling**

In SEM, evaluation of model fit is more complex than in statistical approaches based on variables measured without error. Because there is no single statistical significance test that identifies a correct model (i.e. if the model is plausible) given the sample data, it is necessary to take multiple criteria into consideration and to evaluate model fit based on various measures simultaneously. For each estimation, many goodness-of-fit indices are provided to judge whether the model is consistent with the empirical data. Reporting them all would be a hindrance to the interpretation of model validity, though many studies suggest that the CFI, RMSEA and SRMR indices should be considered alongside the chi-square statistic. (1–4)

1. **Measurement Invariance**

CFA can also be used to test construct validity, thus ensuring that the model is invariant across multiple groups (i.e. gender, ethnicity, age group) as well as whether group comparisons of sample estimates reflect true group differences and are not contaminated by group-specific attributes that are unrelated to the construct of interest (5). A model should pass at least metric invariance testing if we are to be reasonably confident that factors are consistent across sub-groups within the population. Metric invariance occurs when respondents across groups attribute the same meaning to the latent construct under study. This is tested by constraining factor loadings to be equal across groups while allowing intercepts to vary. A model in which the factor loadings are constrained in this way yet fits equally as well as the unconstrained model is metric invariant (5,6).

For our analysis each individual factor was then modelled separately (i.e. four separate CFA models). Each of these individual CFA models achieved excellent goodness of fit scores and were found to be metric invariant across age group and residential area type (as shown in Table S1).

**Table S1: Measurement invariance for masculinity factors**

| **Factor** | **Invariance level** | **df** | **CFI** | **TLI** | **RMSEA** | **SRMR** | **CHI2** |
| --- | --- | --- | --- | --- | --- | --- | --- |
| Community Status | Standard | 2 | 1.00 | 1.00 | 0.01 | 0.03 | 2.40 |
|  | Metric (age group) | 17 | 1.00 | 1.01 | 0.00 | 0.06 | 14.98 |
|  | Metric (area type) | 17 | 0.96 | 0.94 | 0.03 | 0.08 | 31.40 |
| Sex Drive | Standard | 2 | 0.97 | 0.92 | 0.04 | 0.05 | 10.86 |
|  | Metric (age group) | 17 | 0.96 | 0.95 | 0.03 | 0.07 | 29.45 |
|  | Metric (area type) | 17 | 0.94 | 0.92 | 0.04 | 0.07 | 39.23 |
| Anti-femininity | Standard | 2 | 0.96 | 0.89 | 0.03 | 0.03 | 8.82 |
|  | Metric (age group) | 17 | 1.00 | 1.00 | 0.00 | 0.04 | 16.61 |
|  | Metric (area type) | 17 | 0.90 | 0.86 | 0.04 | 0.06 | 37.66 |
| Toughness | Standard | 2 | 1.00 | 0.99 | 0.03 | 0.02 | 6.29 |
|  | Metric (age group) | 17 | 0.99 | 0.99 | 0.02 | 0.05 | 24.32 |
|  | Metric (area type) | 17 | 0.96 | 0.94 | 0.03 | 0.08 | 31.40 |

**Bibliography**

1. Hu, Li-tze, and Peter M. Bentler. “Cutoff Criteria for Fit Indexes in Covariance Structure Analysis: Conventional Criteria versus New Alternatives.” *Structural Equation Modeling: A Multidisciplinary Journal* 6, no. 1 (January 1, 1999): 1–55.
2. Maccallum, Robert C., Michael W. Browne, and Hazuki M. Sugawara. “Power Analysis and Determination of Sample Size for Covariance Structure Modeling.” *Psychological Methods* 1, no. 2 (June 1, 1996): 130–49.
3. Kline, Rex. *Principles and Practice of Structural Equation Modeling*. Guilford Press, 2005.
4. Yu, Ching-Yun. “Evaluating Cutoff Criteria of Model Fit Indices for Latent Variable Models with Binary and Continuous Outcomes.” University of California, n.d.
5. Gregorich, Steven E. “Do Self-Report Instruments Allow Meaningful Comparisons Across Diverse Population Groups? Testing Measurement Invariance Using the Confirmatory Factor Analysis Framework.” *Medical Care* 44, no. 11 Suppl 3 (November 2006): S78–94.
6. Milfont, Taciano L., and Ronald Fischer. “Testing Measurement Invariance across Groups: Applications in Cross-Cultural Research.” *International Journal of Psychological Research* 3, no. 1 (June 18, 2015): 111–30.
